# Prognostic effect of stress hyperglycemia ratio on patients with severe aortic stenosis receiving transcatheter aortic valve replacement: a prospective cohort study

**DOI:** 10.1186/s12933-024-02160-y

**Published:** 2024-02-16

**Authors:** Xiangming Hu, Dejing Feng, Yuxuan Zhang, Can Wang, Yang Chen, Guannan Niu, Zheng Zhou, Zhenyan Zhao, Hongliang Zhang, Moyang Wang, Yongjian Wu

**Affiliations:** 1 Department of Cardiology, Fuwai Hospital, National Center for Cardiovascular Diseases, Chinese Academy of Medical Sciences and Peking Union Medical College, Beijing, China; 2 Department of Cardiology, Peking University People’s Hospital, Beijing, China

**Keywords:** Stress hyperglycemia ratio, Severe aortic stenosis, Transcatheter aortic valve replacement

## Abstract

**Background:**

Stress hyperglycemia ratio (SHR) has recently been recognized as a novel biomarker that accurately reflects acute hyperglycemia status and is associated with poor prognosis of heart failure. We evaluated the relationship between SHR and clinical outcomes in patients with severe aortic stenosis receiving transcatheter aortic valve replacement (TAVR).

**Methods:**

There were 582 patients with severe native aortic stenosis who underwent TAVR consecutively enrolled in the study. The formula used to determine SHR was as follows: admission blood glucose (mmol/L)/(1.59×HbA_1c_[%]–2.59). The primary endpoint was defined as all-cause mortality, while secondary endpoints included a composite of cardiovascular mortality or readmission for heart failure, and major adverse cardiovascular events (MACE) including cardiovascular mortality, non-fatal myocardial infarction, and non-fatal stroke. Multivariable Cox regression and restricted cubic spline analysis were employed to assess the relationship between SHR and endpoints, with hazard ratios (HRs) and 95% confidence intervals (CIs).

**Results:**

During a median follow-up of 3.9 years, a total of 130 cases (22.3%) of all-cause mortality were recorded. Results from the restricted cubic spline analysis indicated a linear association between SHR and all endpoints (p for non-linearity > 0.05), even after adjustment for other confounding factors. Per 0.1 unit increase in SHR was associated with a 12% (adjusted HR: 1.12, 95% CI: 1.04–1.21) higher incidence of the primary endpoint, a 12% (adjusted HR: 1.12, 95% CI: 1.02–1.22) higher incidence of cardiovascular mortality or readmission for heart failure, and a 12% (adjusted HR: 1.12, 95% CI: 1.01–1.23) higher incidence of MACE. Subgroup analysis revealed that SHR had a significant interaction with diabetes mellitus with regard to the risk of all-cause mortality (p for interaction: 0.042). Kaplan-Meier survival analysis showed that there were significant differences in the incidence of all endpoints between the two groups with 0.944 as the optimal binary cutoff point of SHR (all log-rank test: *p* < 0.05).

**Conclusions:**

Our study indicates linear relationships of SHR with the risk of all-cause mortality, cardiovascular mortality or readmission for heart failure, and MACE in patients with severe aortic stenosis receiving TAVR after a median follow-up of 3.9 years. Patients with an SHR exceeding 0.944 had a poorer prognosis compared to those with lower SHR values.

**Supplementary Information:**

The online version contains supplementary material available at 10.1186/s12933-024-02160-y.

## Background

Aortic stenosis (AS) is a common valvular heart disease that significantly worsens with age and is associated with a grim prognosis [1, 2]. According to 2019 Global Disease Burden estimates, there were 9.4 million patients with calcific aortic valve disease, resulting in 130,000 deaths, underscoring the growing imperative for effective disease management [3]. Transcatheter aortic valve replacement (TAVR) has emerged as a pivotal therapeutic approach for severe AS, offering benefits over surgical aortic valve replacement (SAVR) such as reduced trauma, lower risk, and comparable or superior long-term outcomes [4]. As TAVR procedures are refined and indications expand to include patients with low surgical risk, enhancing prognostic evaluation preoperatively becomes paramount. Due to the minimally invasive nature of TAVR, traditional comorbidities and serological markers have shown limited utility in predicting the prognosis of such patients [5–10]. There is an immediate need for innovative, accessible clinical predictors to better ascertain the prognostic outcomes for patients undergoing TAVR.

Previous studies have suggested that abnormalities in glucose metabolism exacerbated cardiovascular complications, particularly in patients with severe AS [11]. Furthermore, These metabolic disturbances are also independently prognostic of mortality following SAVR [12]. High levels of admission blood glucose (ABG) represent a state of metabolic instability in response to the disease and correlate with negative outcomes across a spectrum of diseases [13, 14]. Nonetheless, ABG might not accurately represent acute hyperglycemia, as it is potentially confounded by chronic glycemic control. In recent years, the introduction of the stress hyperglycemia ratio (SHR) has garnered attention as it represents a true hyperglycemic status [15]. Extensive research has validated the prognostic significance of SHR in various cardiovascular diseases, including mitral regurgitation, acute coronary syndrome, and heart failure [16–18]. These studies have demonstrated that elevated SHR was associated with adverse cardiovascular outcomes. However, to date, there have been no studies investigating the impact of SHR on patients with severe AS undergoing TAVR.

Given the potential of SHR as an indicator of perioperative stress response, exploring its correlation with outcomes in severe AS patients undergoing TAVR could be helpful in managing such disease. Therefore, this study aims to assess the prognostic value of SHR in predicting adverse events after TAVR procedure in patients with severe AS.

## Methods

### Study design and population

This study is designed as a prospective cohort study that consecutively included a total of 593 patients who underwent TAVR at Fuwai Hospital from September 2012 to December 2021 (Additional file 1: Fig. S1). Inclusion criteria were: (1) age ≥ 18 years, and (2) patient with severe aortic stenosis treated with TAVR. Exclusion criteria were: (1) patients who received valve-in-valve (TAVR-in-SAVR/TAVR-in-TAVR) treatment, and (2) patients without information about ABG and hemoglobin A1c (HbA_1c_) to calculate SHR. Experienced echocardiographers conducted echocardiographic assessments, following the American Society of Echocardiography Guidelines [19]. The diagnosis of severe AS was based on the combination of three criteria: aortic valve area ≤ 1.0 cm^2^, peak aortic jet velocity ≥ 4 m/s, or mean aortic valve gradient ≥ 40 mmHg [20]. Additional indices obtained from the echocardiogram included left ventricular ejection fraction (LVEF), left atrial diameter, left ventricular end-diastolic dimension, moderate to severe mitral regurgitation, and post-operative perivalvular leakage.

The TAVR treatment decision for severe AS was determined after a multidisciplinary team discussion preoperatively and discussed with the patient and their family among all patients, accounting for age, estimated life expectancy, comorbidities, anatomical and procedural characteristics, feasibility of vascular access, the risks of operation, bioprosthetic valve durability, and the long-term outcome. TAVR procedures were performed according to standard clinical practice [21, 22]. The sizing of the prosthetic valve was based on preoperative computerized tomography measurements and the manufacturer’s recommendations.

The study adhered to the Helsinki Declaration and received approval from the Ethics Review Committee of Fuwai Hospital, National Center for Cardiovascular Diseases (Approval No. 2020 − 1290). Written informed consent was obtained from all patients.

### Covariates

Age, sex, height, weight, smoking status, comorbidities, physical examination, blood-based cardiometabolic indicators, periprocedural condition and medication were considered as covariates in analysis. Body mass index (BMI) was calculated as weight (kg)/(height^2^[m]). All comorbidities were defined based on ICD-10 codes according to medical diagnosis. In addition to medical history, the diagnosis of diabetes mellitus also be determined by the patient’s currently or previously use of oral hypoglycemic agents or insulin, or HbA_1c_ > 6.5%. Stroke included both ischemic and hemorrhagic strokes. Blood tests were conducted in the quality-controlled laboratory at Fuwai Hospital. Due to variations in cardiac troponin I (cTnI) units, cTnI levels were expressed as cTnI ratio (cTnI/upper limit of normal). Renal function was estimated using the estimated glomerular filtration rate (eGFR) calculated using the CKD Epidemiology Collaboration equation [23].

### Exposure

ABG levels were detected using the LABOSPECT 008 system (Hitachi, Tokyo, Japan) at the time of hospital admission. The level of HbA_1c_ was measured by high-performance liquid chromatography (Tosoh G8 HPLC Analyzer, Tosoh Bioscience, Tokyo, Japan). SHR was calculated according to the following formula: ABG (mmol/L)/(1.59×HbA_1c_ [%]–2.59) [15], reflecting the condition at the time of admission.

### Endpoint and follow-up

The primary endpoint was all-cause mortality, while secondary endpoints included a composite of cardiovascular mortality or readmission for heart failure, and major adverse cardiovascular events (MACE) including cardiovascular mortality, non-fatal myocardial infarction, and non-fatal stroke. Clinical events were defined according to the Valve Academic Research Consortium-2 (VARC-2) criteria [24] and confirmed by reviewing the medical records. All patients were followed up at 1 month, 3 months, 6 months, and 1 year after discharge, and subsequently annually through telephone interviews or outpatient visits.

### Data collection

Clinical medical information was recorded in an electronic data collection system and subjected to double verification. Baseline demographic and clinical treatment data for all patients were prospectively collected. Medication details upon discharge were documented.

### Statistical analyses

The patients were divided into four groups based on quartiles of SHR. Baseline data comparisons were conducted using analysis of variance (ANOVA) for normally distributed data, Kruskal-Wallis *H* test for skewed data, and chi-square test/Fisher’s exact test for categorical variables to determine significant differences among the four groups. Linear regression analysis and Wald chi-square tests were conducted to calculate p-values for trend per quartile increase of SHR.

Cox regression models were employed to assess the independent associations between SHR and the incidence of different endpoints, using hazard ratios (HRs) with 95% confidence intervals (CIs). All regression models assessed the proportionality hazard assumption, and the results were satisfactory. Because the SHR value was too small, we extend it 10 times, labeling it as per 0.1 unit change in the Cox regression analysis. Potential covariates that showed clinical relevance or significance in the baseline comparisons without collinearity (Additional file 1: Tables S1–S6) were considered in the multivariate models. Two models were established: Model 1, unadjusted; Model 2, multivariate adjusted. To explore the dose-response relationship between SHR and the incidence of different endpoints, restricted cubic spline (RCS) functions based on Cox regression model were conducted, adjusting for covariates in Model 2. Four knots were set at the 5th, 35th, 65th, and 95th percentiles in the RCS curve. The SHR value at HR = 1 in the RCS curve was taken as the reference value. The optimal binary cutoff point of SHR for primary endpoint was selected using the maximally selected rank statistics method to distinguish between the high and low SHR groups [25]. Event-free survival probabilities were estimated through Kaplan-Meier survival analyses and log-rank tests.

Subgroup analysis was performed to investigate the effect of SHR on the incidence of different endpoints in pre-specified and exploratory subgroups, including age (< / ≥ 75 years), sex (male/female), BMI (< / ≥ 24 kg/m^2^), diabetes mellitus (yes/no), hypertension (yes/no), coronary artery disease (yes/no), chronic heart failure (yes/no), and LVEF (< / ≥ 50%) subgroups. Likelihood ratio tests were conducted to examine modifications and interactions between subgroups. Several sensitivity analyses were carried out to assess the robustness of the results. Firstly, stepwise covariate selection was applied to all baseline variables for different endpoints, and the results were reported as Model 3. Secondly, a Cox logistic least absolute shrinkage and selection operator (LASSO) regression model was used for covariate selection of all baseline variables for different endpoints, with SHR included as a penalty variable to account for collinearity effects, and the results were reported as Model 4 [26]. Thirdly, landmark analyses were performed by excluding patients with an endpoint that occurred within 30-day or 1 year of discharge because these patients may be severely ill and potentially have confounding high SHR values and high mortality. Finally, the analysis was repeated after excluding patients with anemia (hemoglobin < 100 g/L) or severe renal dysfunction (eGFR < 30 ml/min/1.73m^2^).

The proportion of missing data in the sample did not exceed 8%. The last observation carried forward method, as well as means and medians, were employed to impute the missing data. A two-tailed p-value < 0.05 was considered statistically significant. All analyses were performed using Stata 15.0 (StataCorp LLC, College Station, TX, USA) and R version 4.0.2 (The R Project for Statistical Computing, Vienna, Austria).

## Results

### Baseline characteristics

The comparison of baseline information based on SHR quartiles (Q1, 0.66 ± 0.06; Q2, 0.77 ± 0.02; Q3, 0.87 ± 0.03; Q4, 1.14 ± 0.25) is presented in Table 1. The average age of the patients was 75.5 (standard deviation: 7.4), and males constituted 58.4% of the cohort. Patients in the extreme SHR quartiles (Q1 and Q4) exhibited a higher prevalence of diabetes mellitus and chronic kidney disease, coupled with lower initial blood pressure, lipid concentrations, and LVEF compared to those in the intermediate quartiles (Q2 and Q3). The median of EuroSCORE II among all patients was 2.96% (interquartile range [IQR]: 1.85–5.12%), with the highest EuroSCORE II being in the Q4 quartile of SHR, at 3.76% (IQR: 2.20–6.59%). Patients in the Q1 of SHR had the lowest levels of hemoglobin and albumin, while those in the Q4 of SHR had the highest levels of N-terminal pro-brain natriuretic peptide (NT-proBNP). The majority of patients received either local anesthesia or conscious sedation, were performed intervention via a femoral artery approach, and were implanted with a self-expanding valve. The overall incidence of moderate to severe paravalvular leak was observed at 2.7%. The incidence of permanent pacemaker implantation was the highest in the Q1 of SHR (10.9%).

**Table 1:** Baseline information

	All (*n* = 582)	Q1 (*n* = 146)	Q2 (*n* = 145)	Q3 (*n* = 146)	Q4 (*n* = 145)	P	P for trend
SHR	0.85 ± 0.20	0.66 ± 0.06	0.77 ± 0.02	0.87 ± 0.03	1.14 ± 0.25	< 0.001	< 0.001
Demographics and medical history							
Age, years	75.50 ± 7.44	75.82 ± 7.56	76.52 ± 6.84	74.89 ± 7.09	74.76 ± 8.14	0.143	0.081
Male, %	340 (58.42%)	83 (56.85%)	84 (57.93%)	80 (54.79%)	93 (64.14%)	0.407	0.307
BMI*, kg/m^2^	23.59 ± 3.65	23.54 ± 3.93	23.71 ± 3.51	23.86 ± 3.33	23.23 ± 3.80	0.496	0.568
EuroSCORE II*, %	2.96 (1.85–5.12)	3.19 (1.94–5.43)	2.48 (1.79–3.70)	2.82 (1.73–4.40)	3.76 (2.20–6.59)	< 0.001	0.004
NYHA class ≥ III, %	436 (74.91%)	116 (79.45%)	102 (70.34%)	107 (73.29%)	111 (76.55%)	0.305	0.740
Smoking status, %						0.062	0.292
Never	352 (60.48%)	79 (54.11%)	89 (61.38%)	101 (69.18%)	83 (57.24%)		
Ex-smoker	175 (30.07%)	53 (36.30%)	37 (25.52%)	35 (23.97%)	50 (34.48%)		
Current	55 (9.45%)	14 (9.59%)	19 (13.10%)	10 (6.85%)	12 (8.28%)		
Hypertension, %	364 (62.54%)	94 (64.38%)	95 (65.52%)	86 (58.90%)	89 (61.38%)	0.644	0.384
Hyperlipemia, %	362 (62.20%)	87 (59.59%)	96 (66.21%)	89 (60.96%)	90 (62.07%)	0.680	0.901
Coronary heart disease, %	251 (43.13%)	62 (42.47%)	66 (45.52%)	57 (39.04%)	66 (45.52%)	0.638	0.885
Previous myocardial infarction, %	46 (7.90%)	13 (8.90%)	10 (6.90%)	9 (6.16%)	14 (9.66%)	0.655	0.882
Previous coronary revascularization, %	96 (16.49%)	25 (17.12%)	21 (14.48%)	25 (17.12%)	25 (17.24%)	0.904	0.829
Chronic heart failure, %	225 (38.66%)	63 (43.15%)	57 (39.31%)	52 (35.62%)	53 (36.55%)	0.550	0.193
Atrial fibrillation, %	98 (16.84%)	22 (15.07%)	24 (16.55%)	30 (20.55%)	22 (15.17%)	0.561	0.754
Peripheral arterial disease, %	92 (15.81%)	23 (15.75%)	20 (13.79%)	23 (15.75%)	26 (17.93%)	0.817	0.532
Previous valvular intervention, %	14 (2.41%)	4 (2.74%)	2 (1.38%)	2 (1.37%)	6 (4.14%)	0.356	0.465
Previous stroke, %	71 (12.20%)	19 (13.01%)	20 (13.79%)	16 (10.96%)	16 (11.03%)	0.843	0.470
COPD, %	61 (10.48%)	18 (12.33%)	13 (8.97%)	12 (8.22%)	18 (12.41%)	0.520	0.962
Chronic kidney disease, %	50 (8.59%)	21 (14.38%)	9 (6.21%)	7 (4.79%)	13 (8.97%)	0.019	0.087
Diabetes mellitus, %	192 (32.99%)	61 (41.78%)	37 (25.52%)	36 (24.66%)	58 (40.00%)	< 0.001	0.713
Systolic blood pressure, mmHg	128.32 ± 21.54	127.27 ± 22.58	131.57 ± 21.39	130.71 ± 21.31	123.73 ± 20.12	0.007	0.154
Diastolic blood pressure, mmHg	69.46 ± 12.30	68.34 ± 11.82	70.86 ± 11.68	71.06 ± 14.23	67.55 ± 10.96	0.028	0.641
Heart rate, beats/min	75.18 ± 12.75	74.56 ± 12.79	74.59 ± 12.84	74.95 ± 13.32	76.63 ± 12.04	0.461	0.165
Hemoglobin, g/L	127.94 ± 18.45	123.67 ± 17.78	128.25 ± 18.79	131.00 ± 15.87	128.85 ± 20.48	0.006	0.007
Platelet, 10^9^/L	191.09 ± 60.34	186.98 ± 64.69	190.28 ± 57.98	185.75 ± 53.29	201.43 ± 64.02	0.106	0.083
Albumin, g/L	40.18 ± 4.03	39.03 ± 3.79	40.11 ± 3.81	41.11 ± 3.90	40.49 ± 4.36	< 0.001	< 0.001
Uric acid, µmol/L	413.19 ± 138.37	417.36 ± 124.84	397.76 ± 112.44	394.94 ± 136.51	442.81 ± 169.32	0.011	0.155
ABG, mmol/L	6.26 ± 1.99	5.18 ± 1.02	5.55 ± 0.95	6.04 ± 1.23	8.29 ± 2.58	< 0.001	< 0.001
HbA_1C_, %	6.25 ± 0.95	6.60 ± 1.05	6.17 ± 0.78	6.02 ± 0.88	6.21 ± 0.97	< 0.001	< 0.001
HbA_1C_, mmol/mol	44.79 ± 10.34	48.66 ± 11.49	43.89 ± 8.51	42.25 ± 9.57	44.35 ± 10.55	< 0.001	< 0.001
eGFR, ml/min/1.73m^2^	62.99 ± 17.90	61.10 ± 19.73	63.77 ± 15.88	64.83 ± 17.42	62.24 ± 18.30	0.296	0.497
Hs-CRP*, mg/L	1.71 (0.98–4.46)	1.77 (1.00–4.54)	1.67 (1.00–3.81)	1.64 (0.87–3.81)	1.80 (0.89–5.73)	0.599	0.307
Lipoprotein (a)*, mg/L	203.00 (82.68–497.50)	218.31 (86.58–426.74)	154.90 (83.90–583.43)	210.50 (69.74–522.60)	206.00 (85.60–489.46)	0.980	0.453
Triglyceride, mmol/L	1.30 ± 0.84	1.25 ± 0.88	1.37 ± 1.15	1.26 ± 0.57	1.34 ± 0.65	0.516	0.580
Total cholesterol, mmol/L	4.23 ± 1.11	4.20 ± 0.99	4.36 ± 1.27	4.35 ± 1.09	4.00 ± 1.04	0.017	0.131
LDL-C, mmol/L	2.54 ± 0.96	2.57 ± 0.87	2.63 ± 1.17	2.63 ± 0.93	2.35 ± 0.81	0.040	0.060
HDL-C, mmol/L	1.25 ± 0.38	1.22 ± 0.36	1.29 ± 0.38	1.30 ± 0.38	1.20 ± 0.39	0.047	0.598
NT-proBNP*, pg/mL	2071.50 (837.85–5385.25)	2084.00 (978.00–5466.75)	1700.40 (883.90–3374.00)	1717.00 (626.30–4417.75)	3315.10 (1170.00–6817.00)	0.002	0.085
cTnI ratio*	0.74 (0.38–1.50)	0.76 (0.44–1.32)	0.74 (0.32–1.52)	0.65 (0.31–1.31)	0.82 (0.49–2.21)	0.076	0.082
LVEF, %	54.96 ± 13.78	54.04 ± 14.95	57.14 ± 11.97	56.99 ± 13.09	51.66 ± 14.30	0.001	0.158
Left atrial diameter, mm	41.93 ± 6.26	42.12 ± 6.34	41.47 ± 5.44	41.03 ± 6.78	43.10 ± 6.28	0.029	0.699
Left ventricular diastolic diameter, mm	51.61 ± 8.18	52.30 ± 9.03	51.08 ± 6.63	50.81 ± 8.23	52.25 ± 8.58	0.267	0.538
Moderate-to-severe mitral regurgitation, %	122 (20.96%)	30 (20.55%)	24 (16.55%)	32 (21.92%)	36 (24.83%)	0.376	0.229
Insulin	39 (27.66%)	13 (34.21%)	6 (25.00%)	4 (15.38%)	16 (30.19%)		
Periprocedural condition							
Hospital stay before TAVR, days	5.00 (3.00–7.75)	5.00 (3.00–8.00)	5.00 (3.00–7.00)	4.00 (2.00–7.00)	4.00 (2.00–7.00)	0.007	0.120
Bioprosthetic heart valve, %						0.676	0.222
Self-expanding valve	541 (92.96%)	133 (91.10%)	134 (92.41%)	137 (93.84%)	137 (94.48%)		
Balloon-expandable valve	41 (7.04%)	13 (8.90%)	11 (7.59%)	9 (6.16%)	8 (5.52%)		
Access, %						0.072	0.298
Femoral	561 (96.39%)	140 (95.89%)	139 (95.86%)	139 (95.21%)	143 (98.62%)		
Carotid	13 (2.23%)	6 (4.11%)	4 (2.76%)	2 (1.37%)	1 (0.69%)		
Aortic	8 (1.37%)	0 (0.00%)	2 (1.38%)	5 (3.42%)	1 (0.69%)		
Anesthesia, %						0.266	0.085
Local/conscious sedation	348 (59.79%)	96 (65.75%)	85 (58.62%)	88 (60.27%)	79 (54.48%)		
General anesthesia	234 (40.21%)	50 (34.25%)	60 (41.38%)	58 (39.73%)	66 (45.52%)		
Second valve implantation, %	66 (11.34%)	15 (10.27%)	15 (10.34%)	13 (8.90%)	23 (15.86%)	0.249	0.194
Pre-dilatation*, %	541 (92.96%)	130 (89.66%)	132 (91.03%)	141 (96.58%)	137 (94.48%)	0.084	0.054
Post-dilatation*, %	102 (17.53%)	18 (12.41%)	19 (13.10%)	36 (24.66%)	29 (20.00%)	0.016	0.010
Concomitant percutaneous coronary intervention, %	91 (15.64%)	22 (15.07%)	20 (13.79%)	19 (13.01%)	30 (20.69%)	0.264	0.234
Post-procedure mean gradient*, mmHg	12.34 ± 5.72	12.60 ± 6.74	12.13 ± 5.13	13.14 ± 5.76	12.19 ± 6.27	0.505	0.944
Moderate-to-severe perivalvular leakage*, %	16 (2.75%)	3 (2.11%)	2 (1.42%)	4 (2.78%)	7 (4.83%)	0.305	0.194
Permanent pace maker implantation, %	43 (7.39%)	16 (10.96%)	10 (6.90%)	11 (7.53%)	6 (4.14%)	0.171	0.041
Medication							
ACEI/ARB, %	118 (20.27%)	31 (21.23%)	30 (20.69%)	31 (21.23%)	26 (17.93%)	0.879	0.531
β-blocker, %	410 (70.45%)	100 (68.49%)	98 (67.59%)	104 (71.23%)	108 (74.48%)	0.569	0.202
Aspirin, %	433 (74.40%)	100 (68.49%)	108 (74.48%)	116 (79.45%)	109 (75.17%)	0.197	0.121
P2Y12 inhibitor, %	471 (80.93%)	119 (81.51%)	119 (82.07%)	118 (80.82%)	115 (79.31%)	0.940	0.592
Anticoagulant, %	49 (8.42%)	13 (8.90%)	11 (7.59%)	16 (10.96%)	9 (6.21%)	0.509	0.892
Statin, %	439 (75.43%)	105 (71.92%)	115 (79.31%)	112 (76.71%)	107 (73.79%)	0.923	0.477
Glucose-lowering therapy, %						0.625	0.491
Diet control	11 (7.80%)	3 (7.89%)	2 (8.33%)	1 (3.85%)	5 (9.43%)		
Oral hypoglycemic drugs	91 (64.54%)	22 (57.89%)	16 (66.67%)	21 (80.77%)	32 (60.38%)		

Data are means ± SD, median (interquartile range), or n (%)

*missing data: 3 for BMI, 19 for EuroSCORE II, 2 for hs-CRP, 1 for lipoprotein (a), 2 for NT-proBNP, 18 for cTnI ratio, 47 for post-procedure mean gradient, 1 for pre-dilatation, 1 for post-dilatation and 15 for moderate-to-severe perivalvular leakage

*SHR* stress hyperglycemia ratio, *BMI* body mass index, *COPD* chronic obstructive pulmonary disease, *EuroSCORE II* European system for cardiac operative risk evaluation, *ABG* admission blood glucose, *HbA*_*1C*_ glycated hemoglobin A1c, *Hs-CRP* high sensitivity C reactive protein, *LDL-C* low-density lipoprotein cholesterol, *HDL-C* high-density lipoprotein cholesterol, *NT-proBNP* N-terminal brain natriuretic peptide, *cTnI* cardiac troponin I, *LVEF* left ventricular ejection fraction, *NYHA* New York Heart Association, *ACEI* angiotensin-converting enzyme inhibitors, *ARB* angiotensin receptor blockers, *TAVR* transcatheter aortic valve replacement

### Stress hyperglycemia ratio and endpoint﻿s

**Fig. 1:**
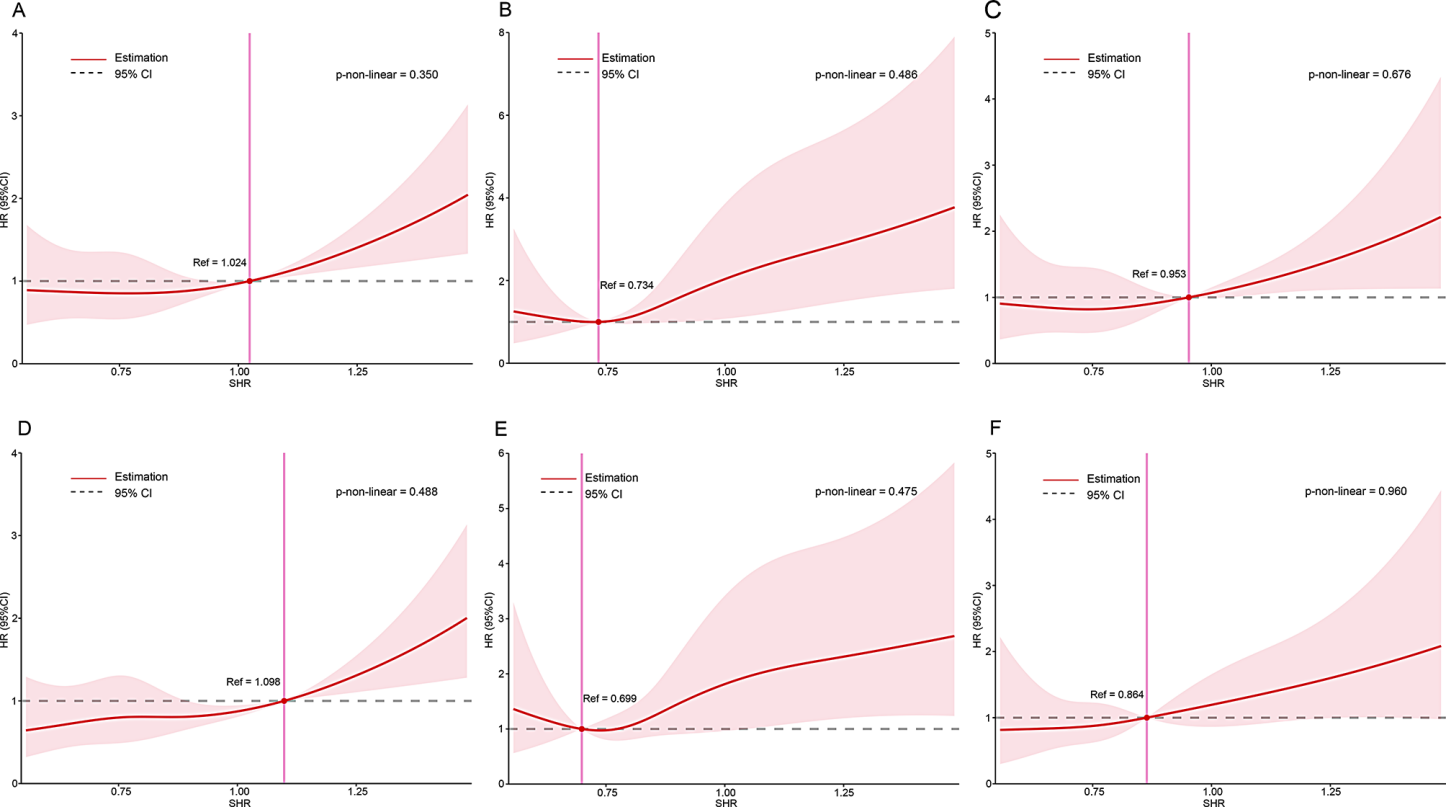
Association of SHR with different endpoints among patients who treated with TAVR. **A**: All-cause mortality-unadjusted. **B**: cardiovascular mortality or readmission for heart failure-unadjusted. **C**: MACE-unadjusted. **D**: All-cause mortality-adjusted. **E**: Cardiovascular mortality and rehospitalization for heart failure-adjusted. **F**: MACE-adjusted. SHR stress hyperglycemia ratio, HR hazard ratio, CI confidence interval, MACE major adverse cardiovascular events

As presented in Table 2, there was no significant difference in the incidence of in-hospital events across the four groups. During a median follow-up period of 3.9 years (IQR: 2.6–5.2), there were 130 (22.3%) cases of all-cause mortality, 61 (10.4%) cases of cardiovascular mortality or readmission for heart failure, and 64 (10.9%) cases of MACE. The dose-response curves between SHR and different endpoints are shown in Fig. 1. There was a linear relationship between SHR and the incidence of different endpoints (all p-values for non-linear: > 0.05). As shown in Table 3, in the fully adjusted Cox regression model, per 0.1 unit increase in SHR was associated with a 12% (adjusted HR: 1.12, 95% CI: 1.04–1.21) increased risk for all-cause mortality, a 12% (adjusted HR: 1.12, 95% CI: 1.02–1.22) increased risk of cardiovascular mortality or readmission for heart failure, and a 12% (adjusted HR: 1.12, 95%CI: 1.01–1.23) increased risk of MACE.

**Table 2:** In-hospital adverse events

	Q1 (*n* = 146)	Q2 (*n* = 145)	Q3 (*n* = 146)	Q4 (*n* = 145)	P	P for trend
Death	2 (1.37%)	0 (0.00%)	2 (1.37%)	3 (2.07%)	0.432	0.396
Non-fatal stroke	0 (0.00%)	2 (1.38%)	1 (0.68%)	2 (1.38%)	0.524	0.316
Non-fatal myocardial infarction	1 (0.68%)	1 (0.69%)	0 (0.00%)	1 (0.69%)	0.799	0.795
Bleeding	0 (0.00%)	2 (1.38%)	2 (1.37%)	1 (0.69%)	0.528	0.548

**Table 3:** HRs (95% CIs) for different endpoints of SHR

	Per 0.1 increase in SHR	High vs. low SHR*
All-cause mortality		
Model 1	1.11 (1.04–1.19)	1.63 (1.12–2.38)
Model 2	1.12 (1.04–1.21)	1.50 (1.01–2.23)
Model 3	1.19 (1.11–1.28)	1.97 (1.33–2.92)
Model 4	1.18 (1.10–1.27)	1.96 (1.32–2.92)
Cardiovascular mortality or readmission for heart failure		
Model 1	1.17 (1.07–1.27)	2.24 (1.32–3.79)
Model 2	1.12 (1.02–1.22)	1.79 (1.04–3.08)
Model 3	1.26 (1.14–1.39)	3.14 (1.76–5.59)
Model 4	1.14 (1.05–1.25)	2.51 (1.47–4.29)
MACE		
Model 1	1.13 (1.02–1.24)	1.76 (1.03–3.01)
Model 2	1.12 (1.01–1.23)	1.79 (1.03–3.12)
Model 3	1.10 (1.00–1.21)	1.85 (1.07–3.20)
Model 4	1.15 (1.03–1.28)	1.88 (1.08–3.27)

Model 1: unadjusted

Model 2: adjusted for age, sex, COPD, diabetes mellitus, EuroSCORE II, chronic kidney disease, albumin, hs-CRP, HDL-C, moderate-to-severe mitral regurgitation, left atrial diameter and moderate-to-severe perivalvular leakage in all-cause mortality, and adjusted for diabetes mellitus, coronary heart disease, EuroSCORE II, moderate-to-severe mitral regurgitation, left atrial diameter and moderate-to-severe perivalvular leakage in cardiovascular mortality or readmission for heart failure, and adjusted for previous stroke, coronary heart disease, diabetes mellitus, atrial fibrillation, EuroSCORE II and albumin in MACE

Model 3: adjusted for covariates based on stepwise Cox regression model

Model 4: adjusted for covariates based on Cox LASSO regression model

*The dichotomy of SHR (0.944) was determined by the maximally selected rank statistics

*SHR* stress hyperglycemia ratio, *HR* hazard ratio, *CI* confidence interval, *MACE* major adverse cardiovascular events

**Fig. 2:**
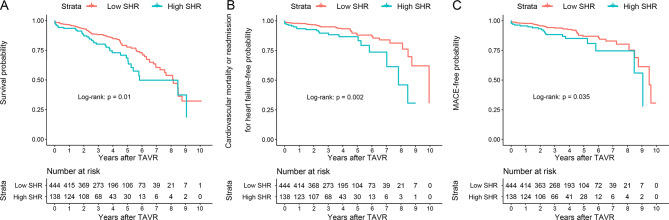
Kaplan-Meier survival analyses for different endpoints among patients who treated with TAVR. **A**: All-cause mortality. **B**: cardiovascular mortality or readmission for heart failure. **C**: MACE. The dichotomy of SHR (0.944) was determined by the maximally selected rank statistics. SHR stress hyperglycemia ratio, MACE major adverse cardiovascular events, TAVR transcatheter aortic valve replacement

As shown in Fig. 2, when patients were stratified into two groups based on the optimal binary cutoff point of SHR (0.944), Kaplan-Meier survival analysis demonstrated significant differences in the incidence of all-cause mortality, cardiovascular mortality or readmission for heart failure, and MACE during the follow-up, with higher rate in the high SHR group (all p-values for log-rank test: < 0.05). Compared to low SHR patients, the adjusted HRs of high SHR patients were 1.50 (95% CI: 1.01–2.23) for all-cause mortality, 1.79 (95% CI: 1.04–3.08) for cardiovascular mortality or readmission for heart failure, and 1.79 (95% CI: 1.03–3.12) for MACE, respectively (Table 3).

### Subgroup analyses

The relationship between SHR and the incidence of different endpoints in most subgroups was similar to the main findings (Additional file 1: Figs. S2–S4). Effect modification was observed between SHR and diabetes mellitus regarding the primary endpoint (p for interaction: 0.042). The association between SHR and primary endpoint was significant in patients with diabetes mellitus (HR: 1.20, 95% CI: 1.10–1.31).

### Sensitivity analyses

Sensitivity analyses further confirmed the robust of the results. After adjusting for covariates selected through stepwise regression and Cox LASSO regression, the relationship between SHR and different endpoints remained consistent with the main results (Table 3). Among the survivors 30 days post-discharge, SHR was significantly associated with all-cause mortality (*p* = 0.017), cardiovascular mortality or readmission for heart failure (*p* = 0.002), and showed a marginal association with MACE (Additional file 1: Fig. S5). The significant association between SHR and all-cause mortality was still observed after excluding patients with an endpoint that occurred within 1 year of discharge (*p* = 0.034), while the association exhibited a marginal positive effect regarding the secondary endpoints (Additional file 1: Fig. S6). Furthermore, the association between SHR and different endpoints remained statistically significant in the subgroup of excluding patients with anemia or severe renal dysfunction (Additional file 1: Tables 7 and 8).

## Discussion

In this first study of SHR and adverse cardiovascular outcomes in patients with severe AS received TAVR over a median follow-up of 3.9 years, we found that: (1) SHR was independently associated with all-cause mortality, cardiovascular mortality or readmission for heart failure, and MACE, after adjusting for covariates, and (2) these associations were linear, and (3) the optimal binary cutoff point of SHR for all-cause mortality was 0.944. Our study suggests that SHR, as a simple indicator, can be used to identify patients with severe AS who are at high risk of adverse outcomes, even after TAVR.

Previous research consistently underscores the prognostic relevance of perioperative assessments in patients undergoing TAVR, which should receive attention from clinical physicians [27, 28]. Considering that AS is predominantly an age-related disease, its prevalence is higher among elderly individuals who often exhibit a higher burden of comorbidities, especially cardiometabolic diseases [18, 29]. The significance of stress-related states becomes particularly pronounced in symptomatic patients with severe AS and complex complications. The concept of SHR was originally introduced by Roberts et al. who demonstrated that SHR was a more effective biomarker for stress-induced hyperglycemia than ABG levels [15]. Stress hyperglycemia reflects to some extent the stress state of the disease and the poorer level of blood sugar control, which may be result in cardiac damage. Previous studies on the SHR have largely concentrated on coronary heart disease and heart failure [16–18]. A study found that the SHR exhibited a U-shaped relationship with major adverse cardiovascular and cerebrovascular events over a 2-year follow-up in 5562 patients with acute coronary syndrome who underwent drug-eluting stent implantation [16]. It highlighted the long-term prognostic value of the SHR and proposed a cutoff value of 0.78 for SHR, which was similar to the cutoff value corresponding to the secondary endpoints in our study. Zhou et al. enrolled 1904 patients with acute decompensated heart failure and found that the SHR has been linked to an unfavorable prognosis, especially among patients in the highest quintile of SHR [18]. However, there is a paucity of research focusing on the prognostic value of SHR in valvular heart disease, particularly in cases when valve intervention has already been performed. One study included 874 patients with secondary mitral regurgitation unveiled a linear correlation between elevated SHR levels and an increased risk of heart function deterioration [17]. Our study presented compelling evidence affirming the significant association between SHR and long-term prognosis in patients with severe aortic stenosis undergoing TAVR, marked by different endpoints. These findings are consistent with previous studies. Furthermore, we have defined a cutoff value of SHR that holds prognostic significance to identify high-risk patients, which can be practical and informative.

We found that patients in the highest quartile of SHR exhibited more severe myocardial injury and worse cardiac function compared with those in lowest quartile of SHR, as indicated by increased levels of NT-ProBNP, and cTNI, and reduced LVEF. These findings provide further support for the association between SHR and cardiac damage in severe AS patients. Moreover, the results from our long-term follow-up suggest that detrimental cardiac effects of SHR may persist even after TAVR procedure and manifested in subsequent clinical events. Another finding is the interaction between SHR and diabetes mellitus regarding the endpoint of all-cause mortality. Specifically, the effect of SHR was significant in patients with diabetes mellitus. The applicability of SHR in both diabetic and non-diabetic populations remains a subject of debate [17, 18, 29, 30]. Zhou et al. found that the prognostic value of SHR only presented in those patients with diabetes mellitus [18], which was similar to the modification effect of diabetes mellitus in the primary endpoint found in our study. However, Kojima et al. recruited 6287 subjects with ST-elevation myocardial infarction with a median follow-up of 4.1 years. They found that high levels of SHR were significantly associated with poorer long-term prognosis in patients without diabetes mellitus rather than those with [30]. The observed variability in the effects might be due to disparities in disease spectrum, sample sizes, and the definitions of study endpoints. Since diabetes mellitus and AS share common pathogenic mechanisms that are detrimental to the heart, it is plausible that the impact of SHR in patients with diabetes mellitus can be attributed to the presence of insulin resistance and severe cardiac damage in this subgroup of patients, and hyperglycemia exacerbating the risk of mortality [31, 32]. This association also underscores the potential clinical relevance of monitoring SHR in individuals with diabetes mellitus.

The specific mechanisms underlying the association between SHR and adverse cardiovascular prognosis remain unclear. Stress-induced hyperglycemia is a physiological response of the body to various critical situation aimed at restoring metabolic balance, even in patients without diabetes [33]. In patients with severe aortic stenosis, long-term decreased cardiac output leads to systemic hypoperfusion, which could result in the overactivation of the adrenergic and renin-angiotensin systems, hyperinsulinemia, and pancreatic β-cell dysfunction leading to insulin resistance, all of which are indicative of abnormal glucose metabolism [34]. In our study, it was noted that the patients in the Q4 of SHR had the highest EuroSCORE II, indicating that SHR was closely related to a critical illness state causing stress response. Additionally, insights from animal and human studies emphasized the role of oxidative stress and inflammation [34, 35]. In the present study, it can be observed the Q4 group of SHR had the highest levels of high-sensitivity C-reactive protein. Although the difference was not statistically significant, it implied a potential association between elevated SHR and an inflammatory response. However, there is also an alternative perspective that emphasizes SHR as an evolutionarily conserved adaptive protective mechanism [36]. Although we did observe the “protective” range of SHR regarding the primary endpoint, further research is warranted to delve deeper into the underlying mechanisms.

The SHR serves as a pragmatic and straightforward prognostic marker for identifying high-risk patients. Clinical physicians should pay closer attention to patients with severe AS who is planned to receive TAVR with high levels of SHR, and proactively implement strategies to mitigate the risk of adverse cardiovascular events and enhance survival.

This is the first study focusing on the predictive value of SHR in long-term adverse outcomes of patients with severe aortic stenosis undergoing TAVR. We described the linear relationship between SHR and adverse outcomes. Additionally, we employed different endpoint definitions to comprehensive discuss the prognostic value of SHR. However, this study has some limitations. Firstly, as it is an observational study design, we cannot rule out the potential impact of confounding factors. Nevertheless, we have conducted various sensitivity analyses to confirm the robustness of our results. Furthermore, this study is a single-center study, and the generalizability of the conclusions awaits further confirmation from others. Finally, the majority of patients in this study used self-expandable valves. The impact of SHR in patients with balloon-expandable valves needs to be further investigated in future studies.

## Conclusions

In conclusion, this study demonstrates linear correlations between SHR and all-cause mortality, cardiovascular mortality or readmission for heart failure, and MACE in patients with severe aortic stenosis received TAVR over a median follow-up of 3.9 years. The cut-offf value of SHR for distinguishing poor prognosis was identified as 0.944. These findings suggest that SHR may be useful for risk stratification in patients after TAVR. Future larger-scale, multicenter studies is needed to validate our findings.

### Electronic supplementary material

Below is the link to the electronic supplementary material.

**Supplementary Material 1:** Additional file 1

## Data Availability

Data relating to this study can be appropriately requested from the corresponding author.

## Abbreviations

ABG: Admission blood glucose
ANOVA: Analysis of variance
AS: Aortic stenosis
BMI: Body mass index
CI: Confidence interval
cTnI: Cardiac troponin I
eGFR: Estimated glomerular filtration rate
HbA_1c_: Hemoglobin A1c
HR: Hazard ratios
IQR: Interquartile range
LASSO: Logistic least absolute shrinkage and selection operator
LVEF: Left ventricular ejection fraction
MACE: Major adverse cardiovascular event
NT-proBNP: N-terminal pro brain natriuretic peptide
RCS: Restricted cubic spline
SAVR: Surgical aortic valve replacement
SHR: Stress hyperglycemia ratio
TAVR: Transcatheter aortic valve replacement
VARC-2: Valve Academic Research Consortium-2
