# Supplementary material for: Prognostic effect of stress hyperglycemia ratio on patients with severe aortic stenosis receiving transcatheter aortic valve replacement: a prospective cohort study
Source: Cardiovasc Diabetol. 2024 Feb 16;23:73. doi: 10.1186/s12933-024-02160-y (PMC10870928; doi:10.1186/s12933-024-02160-y)
Supplement: Supplementary file 1 — Supplementary Material 1: Additional file 1 [file 12933_2024_2160_MOESM1_ESM.docx]

Prognostic effect of stress hyperglycemia ratio on patients with severe aortic stenosis receiving transcatheter aortic valve replacement: a prospective cohort study

Supplementary Figure 1 Study flowchart

Supplementary Figure 2 Subgroup analysis of HRs (95% CIs) for all-cause mortality of SHR

Supplementary Figure 3 Subgroup analysis of HRs (95% CIs) for cardiovascular mortality or readmission for heart failure of SHR

Supplementary Figure 4 Subgroup analysis of HRs (95% CIs) for MACE of SHR

Supplementary Figure 5 Landmark survival analyses (30-day) for different endpoints among patients who treated with TAVR according to dichotomy of SHR

Supplementary Figure 6 Landmark survival analyses (1-year) for different endpoints among patients who treated with TAVR according to dichotomy of SHR

Supplementary Table 1 Baseline information on occurrence and absence of all-cause mortality

Supplementary Table 2 Baseline information on occurrence and absence of cardiovascular mortality or readmission for heart failure

Supplementary Table 3 Baseline information on occurrence and absence of MACE

Supplementary Table 4 Univariate analysis for all-cause mortality

Supplementary Table 5 Univariate analysis for cardiovascular mortality or readmission for heart failure

Supplementary Table 6 Univariate analysis for MACE

Supplementary Table 7 HRs (95% CIs) for different endpoints of SHR after excluding those with hemoglobin ＜ 100g/L (n = 564)

Supplementary Table 8 HRs (95% CIs) for different endpoints of SHR after excluding those with eGFR < 30 ml/min/m^2^ (n = 559)

Supplementary Figure 1 Study flowchart


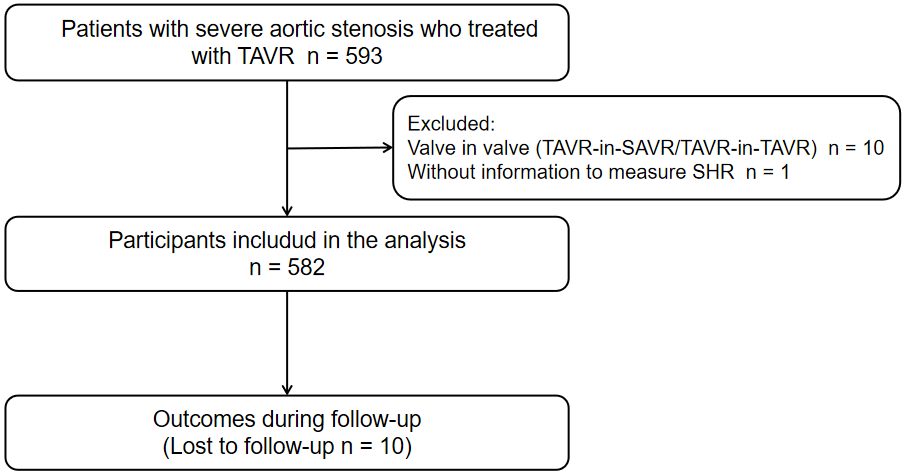


*TAVR* transcatheter aortic valve replacement, *SAVR* surgical aortic valve replacement, *SHR* stress hyperglycemia ratio

Supplementary Figure 2 Subgroup analysis of HRs (95% CIs) for all-cause mortality of SHR


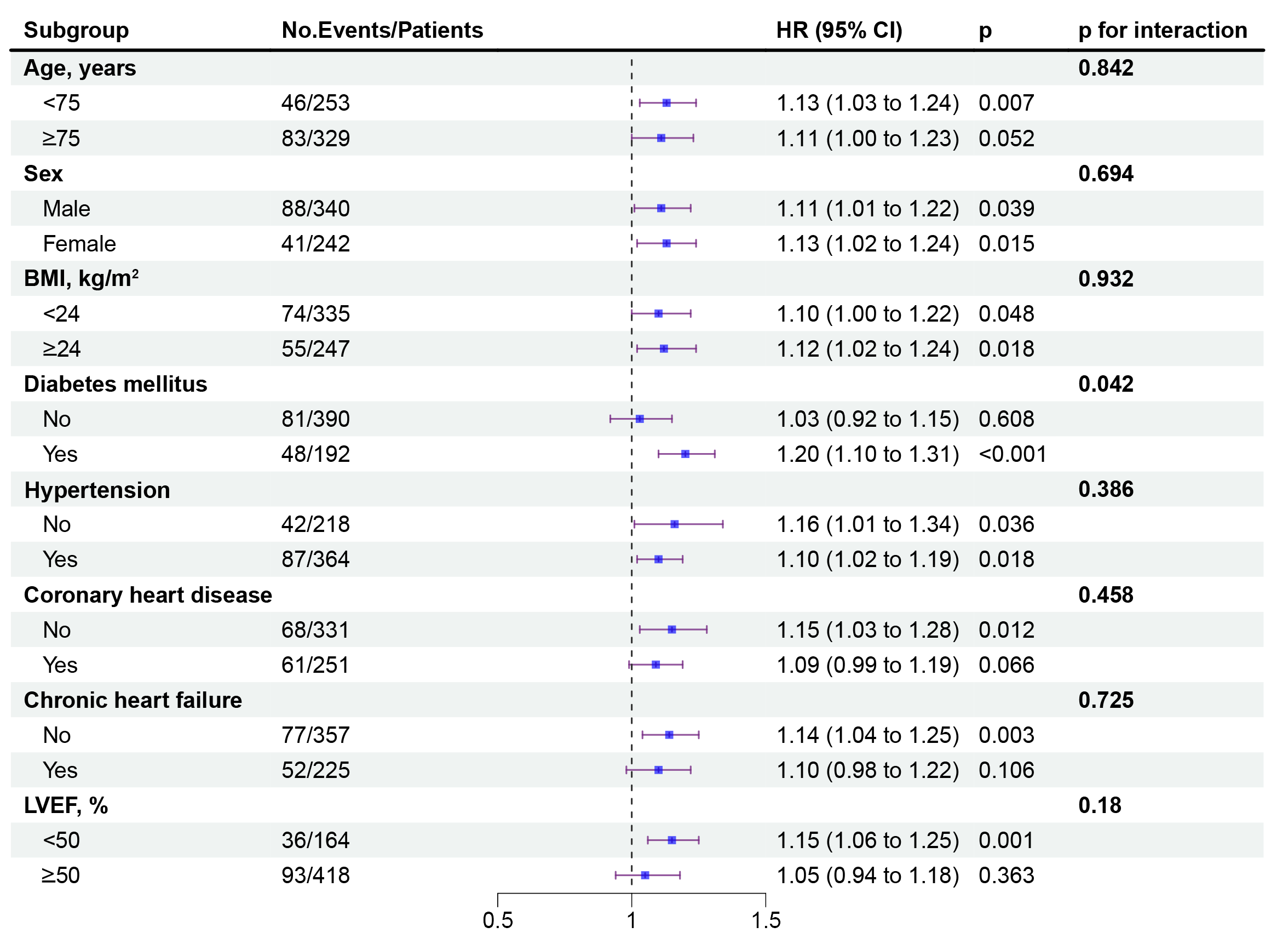


*SHR* stress hyperglycemia ratio, *TAVR* transcatheter aortic valve replacement, *BMI* body mass index, *LVEF* left ventricular ejection fraction, *HR* hazard ratio, *CI* confidence interval

Supplementary Figure 3 Subgroup analysis of HRs (95% CIs) for cardiovascular mortality or readmission for heart failure of SHR


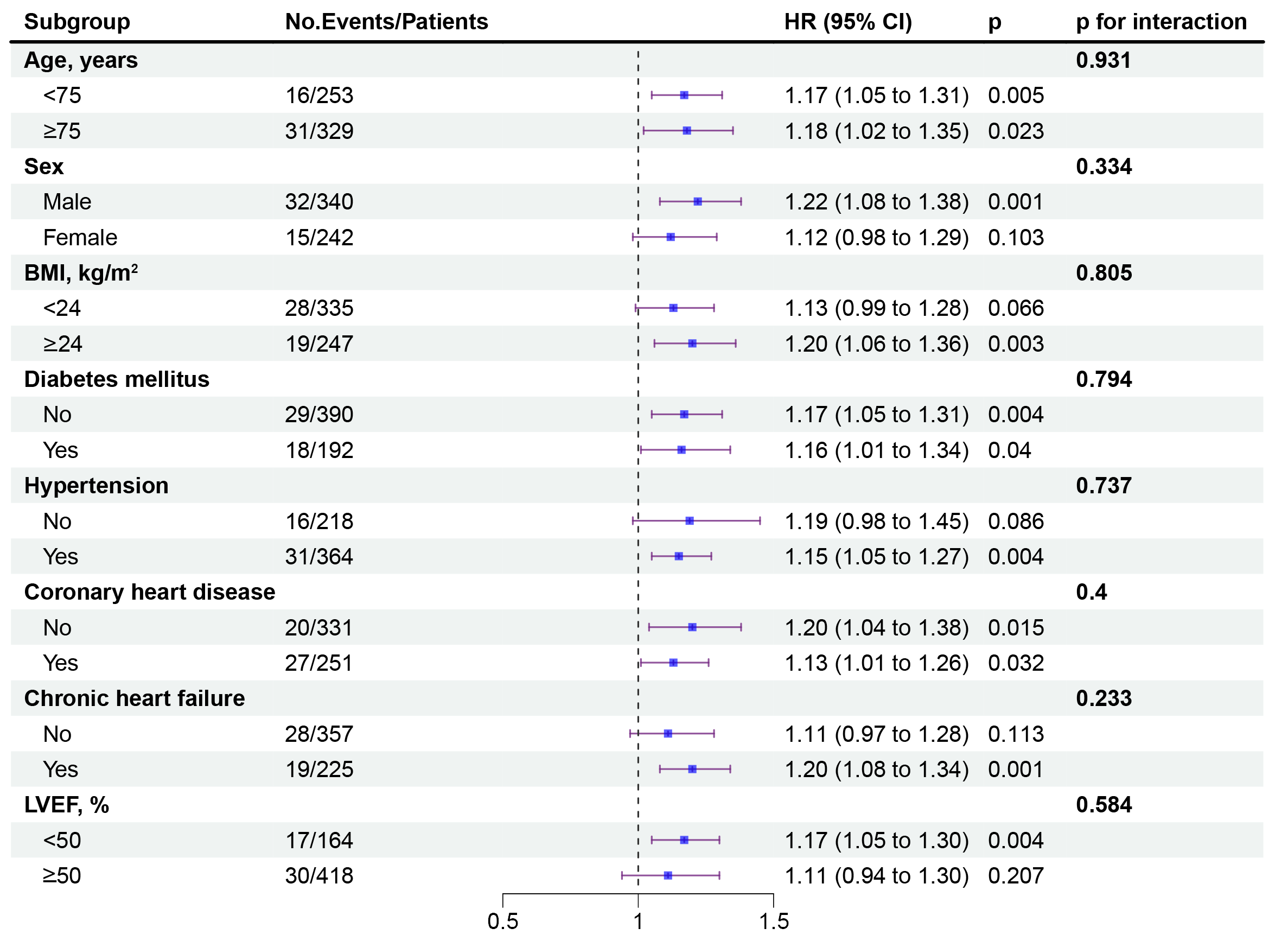


*SHR* stress hyperglycemia ratio, *TAVR* transcatheter aortic valve replacement, *BMI* body mass index, *LVEF* left ventricular ejection fraction, *HR* hazard ratio, *CI* confidence interval

Supplementary Figure 4 Subgroup analysis of HRs (95% CIs) for MACE of SHR


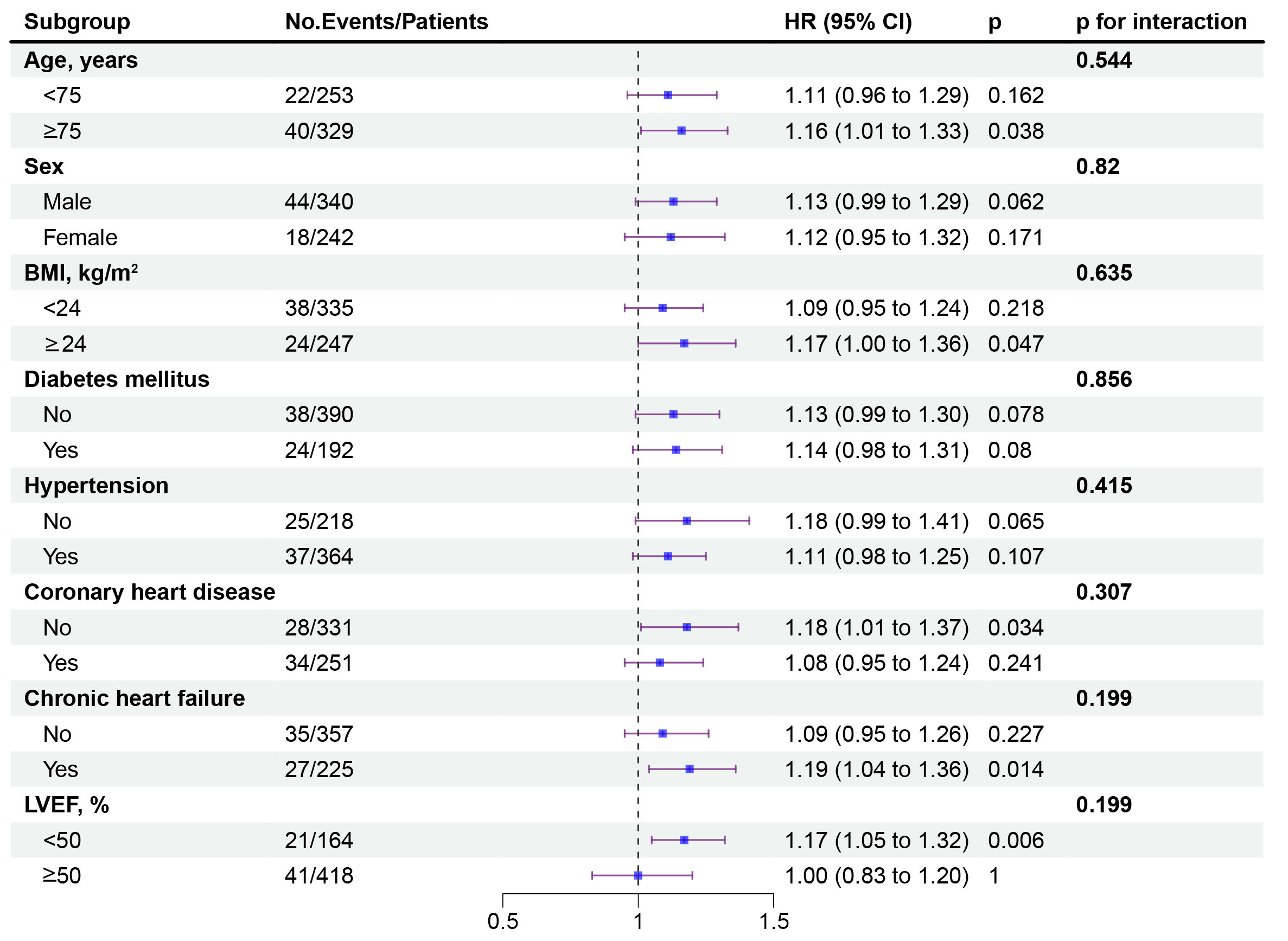


*SHR* stress hyperglycemia ratio, *MACE* major adverse cardiovascular events, *TAVR* transcatheter aortic valve replacement, *BMI* body mass index, *LVEF* left ventricular ejection fraction, *HR* hazard ratio, *CI* confidence interval

Supplementary Figure 5 Landmark survival analyses (30-day) for different endpoints among patients who treated with TAVR according to dichotomy of SHR


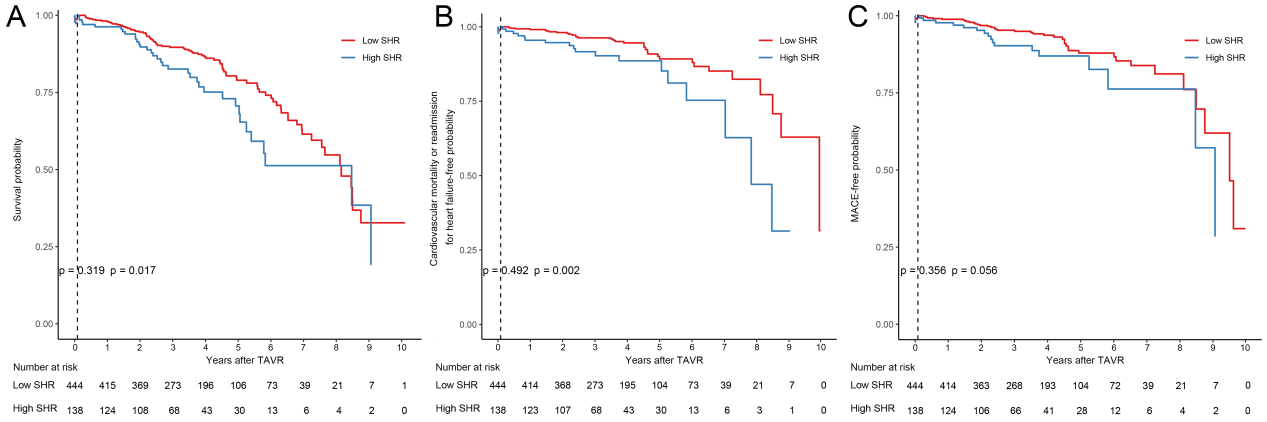


A: All-cause mortality. B: Cardiovascular mortality or readmission for heart failure. C: MACE

*SHR* stress hyperglycemia ratio, *MACE* major adverse cardiovascular events, *TAVR* transcatheter aortic valve replacement

Supplementary Figure 6 Landmark survival analyses (1-year) for different endpoints among patients who treated with TAVR according to dichotomy of SHR


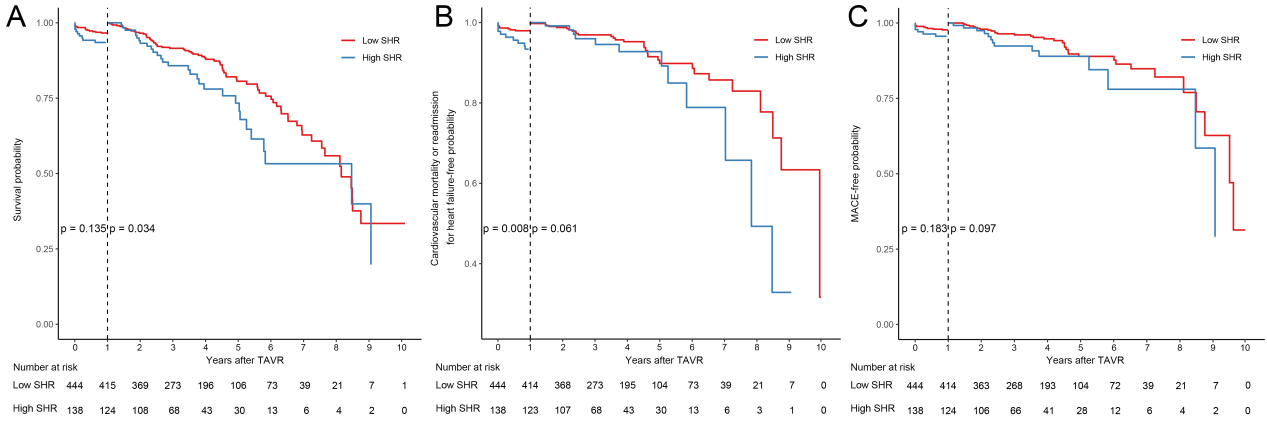


A: All-cause mortality. B: Cardiovascular mortality or readmission for heart failure. C: MACE

*SHR* stress hyperglycemia ratio, *MACE* major adverse cardiovascular events, *TAVR* transcatheter aortic valve replacement

Supplementary Table 1 Baseline information on occurrence and absence of all-cause mortality

|  | No all-cause mortality  (n=452) | All-cause mortality  (n=130) | p value |
| --- | --- | --- | --- |
| Age, years | 75.04 ± 7.64 | 77.10 ± 6.49 | 0.005 |
| Male, % | 251 (55.53%) | 89 (68.46%) | 0.008 |
| BMI, kg/m^2^ | 23.66 ± 3.47 | 23.35 ± 4.21 | 0.408 |
| EuroSCORE II, % | 0.03 (0.02–0.05) | 0.03 (0.02–0.05) | 0.019 |
| NYHA class ≥ III, % | 331 (73.23%) | 105 (80.77%) | 0.081 |
| Smoking status, % |  |  | 0.092 |
| Never | 283 (62.61%) | 69 (53.08%) |  |
| Ex-smoker | 126 (27.88%) | 49 (37.69%) |  |
| Current | 43 (9.51%) | 12 (9.23%) |  |
| Hypertension, % | 276 (61.06%) | 88 (67.69%) | 0.169 |
| Hyperlipemia, % | 287 (63.50%) | 75 (57.69%) | 0.229 |
| Coronary heart disease, % | 189 (41.81%) | 62 (47.69%) | 0.233 |
| Previous myocardial infarction, % | 36 (7.96%) | 10 (7.69%) | 0.919 |
| Previous coronary revascularization, % | 86 (16.10%) | 10 (20.83%) | 0.398 |
| Chronic heart failure, % | 173 (38.27%) | 52 (40.00%) | 0.722 |
| Atrial fibrillation, % | 71 (15.71%) | 27 (20.77%) | 0.174 |
| Peripheral arterial disease, % | 70 (15.49%) | 22 (16.92%) | 0.692 |
| Previous valvular intervention, % | 10 (2.21%) | 4 (3.08%) | 0.571 |
| Previous stroke, % | 49 (10.84%) | 22 (16.92%) | 0.062 |
| COPD, % | 38 (8.41%) | 23 (17.69%) | 0.002 |
| Chronic kidney disease, % | 30 (6.64%) | 20 (15.38%) | 0.002 |
| Diabetes mellitus, % | 144 (31.86%) | 48 (36.92%) | 0.279 |
| Systolic blood pressure, mmHg | 129.09 ± 21.77 | 125.65 ± 20.57 | 0.109 |
| Diastolic blood pressure, mmHg | 69.29 ± 11.87 | 70.03 ± 13.74 | 0.546 |
| Heart rate, beats/min | 75.03 ± 12.64 | 75.72 ± 13.16 | 0.590 |
| Hemoglobin, g/L | 129.10 ± 17.87 | 123.91 ± 19.88 | 0.005 |
| Platelet, 10^9^/L | 191.57 ± 58.03 | 189.44 ± 67.96 | 0.723 |
| Albumin, g/L | 40.61 ± 3.87 | 38.71 ± 4.26 | <0.001 |
| Uric acid, μmol/L | 408.19 ± 137.73 | 430.59 ± 139.71 | 0.104 |
| eGFR, ml/min/1.73m^2^ | 63.57 ± 17.32 | 60.94 ± 19.71 | 0.140 |
| Hs-CRP, mg/L | 1.54 (0.86–3.84) | 2.38 (1.15–8.63) | <0.001 |
| Lipoprotein (a), mg/L | 190.00 (77.00–466.99) | 230.34 (95.98–565.89) | 0.212 |
| Triglyceride, mmol/L | 1.29 ± 0.81 | 1.36 ± 0.95 | 0.394 |
| Total cholesterol, mmol/L | 4.23 ± 1.12 | 4.23 ± 1.08 | 0.975 |
| LDL-C, mmol/L | 2.52 ± 0.97 | 2.63 ± 0.93 | 0.224 |
| HDL-C, mmol/L | 1.28 ± 0.39 | 1.16 ± 0.33 | 0.002 |
| NT-proBNP, pg/mL | 1812.00 (788.00–5189.75) | 2531.05 (1406.43–5961.50) | 0.022 |
| cTnI ratio | 0.71 (0.35–1.50) | 0.75 (0.46–1.46) | 0.297 |
| LVEF, % | 55.00 ± 13.59 | 54.83 ± 14.47 | 0.900 |
| Left atrial diameter, mm | 41.44 ± 6.18 | 43.61 ± 6.28 | <0.001 |
| Left ventricular diastolic diameter, mm | 51.53 ± 8.05 | 51.88 ± 8.63 | 0.688 |
| Moderate-to-severe mitral regurgitation, % | 86 (19.03%) | 36 (27.69%) | 0.032 |
| ACEI/ARB, % | 86 (19.03%) | 32 (24.62%) | 0.162 |
| β-blocker, % | 316 (69.91%) | 94 (72.31%) | 0.598 |
| Aspirin, % | 343 (75.88%) | 90 (69.23%) | 0.126 |
| P2Y12 inhibitor, % | 368 (81.42%) | 103 (79.23%) | 0.576 |
| Anticoagulant, % | 40 (8.85%) | 9 (6.92%) | 0.486 |
| Statin, % | 352 (77.88%) | 87 (66.92%) | 0.011 |
| Glucose-lowering therapy, % |  |  |  |
| Diet control | 6 (5.77%) | 5 (13.51%) | 0.316 |
| Oral hypoglycemic drugs | 69 (66.35%) | 22 (59.46%) |  |
| Insulin | 29 (27.88%) | 10 (27.03%) |  |
| Post-procedure mean gradient, mmHg | 12.44 ± 5.74 | 11.99 ± 5.67 | 0.430 |
| Moderate-to-severe perivalvular leakage, % | 9 (1.99%) | 7 (5.38%) | 0.037 |

*SHR* stress hyperglycemia ratio, *BMI* body mass index, *COPD* chronic obstructive pulmonary disease, *EuroSCORE II* European system for cardiac operative risk evaluation, *Hs-CRP* high sensitivity C reactive protein, *LDL-C* low-density lipoprotein cholesterol, *HDL-C* high-density lipoprotein cholesterol, *NT-proBNP* N-terminal brain natriuretic peptide, *cTnI* cardiac troponin I, *LVEF* left ventricular ejection fraction, *NYHA* New York Heart Association, *ACEI* angiotensin-converting enzyme inhibitors, *ARB* angiotensin receptor blockers.

Supplementary Table 2 Baseline information on occurrence and absence of cardiovascular mortality or readmission for heart failure

|  | No cardiovascular mortality or readmission for heart failure  (n=521) | Cardiovascular mortality or readmission for heart failure (n=61) | p value |
| --- | --- | --- | --- |
| Age, years | 75.31 ± 7.52 | 77.55 ± 6.15 | 0.046 |
| Male, % | 220 (42.23%) | 22 (36.07%) | 0.356 |
| BMI, kg/m^2^ | 23.62 ± 3.54 | 23.32 ± 4.44 | 0.543 |
| EuroSCORE II, % | 0.03 (0.02–0.05) | 0.04 (0.02–0.08) | 0.003 |
| NYHA class ≥ III, % | 386 (74.09%) | 50 (81.97%) | 0.179 |
| Smoking status, % |  |  | 0.589 |
| Never | 316 (60.65%) | 36 (59.02%) |  |
| Ex-smoker | 154 (29.56%) | 21 (34.43%) |  |
| Current | 51 (9.79%) | 4 (6.56%) |  |
| Hypertension, % | 324 (62.19%) | 40 (65.57%) | 0.605 |
| Hyperlipemia, % | 330 (63.34%) | 32 (52.46%) | 0.097 |
| Coronary heart disease, % | 217 (41.65%) | 34 (55.74%) | 0.036 |
| Previous myocardial infarction, % | 38 (7.29%) | 8 (13.11%) | 0.111 |
| Previous coronary revascularization, % | 68 (15.42%) | 28 (19.86%) | 0.216 |
| Chronic heart failure, % | 198 (38.00%) | 27 (44.26%) | 0.342 |
| Atrial fibrillation, % | 81 (15.55%) | 17 (27.87%) | 0.015 |
| Peripheral arterial disease, % | 81 (15.55%) | 11 (18.03%) | 0.615 |
| Previous valvular intervention, % | 9 (1.73%) | 5 (8.20%) | 0.002 |
| Previous stroke, % | 59 (11.32%) | 12 (19.67%) | 0.059 |
| COPD, % | 53 (10.17%) | 8 (13.11%) | 0.478 |
| Chronic kidney disease, % | 43 (8.25%) | 7 (11.48%) | 0.396 |
| Diabetes mellitus, % | 171 (32.82%) | 21 (34.43%) | 0.801 |
| Systolic blood pressure, mmHg | 129.09 ± 21.76 | 121.70 ± 18.38 | 0.011 |
| Diastolic blood pressure, mmHg | 69.36 ± 12.21 | 70.26 ± 13.18 | 0.589 |
| Heart rate, beats/min | 75.19 ± 12.91 | 75.10 ± 11.40 | 0.956 |
| Hemoglobin, g/L | 128.43 ± 18.23 | 123.79 ± 19.95 | 0.063 |
| Platelet, 10^9^/L | 191.63 ± 61.75 | 186.52 ± 46.73 | 0.532 |
| Albumin, g/L | 40.34 ± 3.98 | 38.82 ± 4.24 | 0.005 |
| Uric acid, μmol/L | 413.05 ± 136.16 | 414.45 ± 157.22 | 0.940 |
| eGFR, ml/min/1.73m^2^ | 62.93 ± 18.03 | 63.47 ± 16.88 | 0.823 |
| Hs-CRP, mg/L | 1.68 (0.99–4.33) | 2.08 (1.00–8.68) | 0.340 |
| Lipoprotein (a), mg/L | 202.00 (78.30–489.88) | 236.18 (119.10–553.37) | 0.206 |
| Triglyceride, mmol/L | 1.31 ± 0.87 | 1.24 ± 0.49 | 0.556 |
| Total cholesterol, mmol/L | 4.24 ± 1.12 | 4.17 ± 1.04 | 0.641 |
| LDL-C, mmol/L | 2.53 ± 0.92 | 2.67 ± 1.23 | 0.276 |
| HDL-C, mmol/L | 1.26 ± 0.38 | 1.19 ± 0.33 | 0.181 |
| NT-proBNP, pg/mL | 1901.00 (820.20–5363.00) | 2816.00 (1360.00–5661.00) | 0.067 |
| cTnI ratio | 0.74 (0.38–1.53) | 0.69 (0.37–1.27) | 0.472 |
| LVEF, % | 55.39 ± 13.61 | 51.33 ± 14.75 | 0.029 |
| Left atrial diameter, mm | 41.65 ± 6.19 | 44.28 ± 6.42 | 0.002 |
| Left ventricular diastolic diameter, mm | 51.46 ± 8.05 | 52.84 ± 9.15 | 0.215 |
| Moderate-to-severe mitral regurgitation, % | 103 (19.77%) | 19 (31.15%) | 0.039 |
| ACEI/ARB, % | 106 (20.35%) | 12 (19.67%) | 0.902 |
| β-blocker, % | 374 (71.79%) | 36 (59.02%) | 0.039 |
| Aspirin, % | 393 (75.43%) | 40 (65.57%) | 0.095 |
| P2Y12 inhibitor, % | 426 (81.77%) | 45 (73.77%) | 0.133 |
| Anticoagulant, % | 44 (8.45%) | 5 (8.20%) | 0.947 |
| Statin, % | 398 (76.39%) | 41 (67.21%) | 0.115 |
| Glucose-lowering therapy, % |  |  |  |
| Diet control | 10 (7.81%) | 1 (7.69%) | 0.965 |
| Oral hypoglycemic drugs | 83 (64.84%) | 8 (61.54%) |  |
| Insulin | 35 (27.34%) | 4 (30.77%) |  |
| Post-procedure mean gradient, mmHg | 12.39 ± 5.82 | 11.97 ± 4.82 | 0.591 |
| Moderate-to-severe perivalvular leakage, % | 10 (1.92%) | 6 (9.84%) | <0.001 |

*SHR* stress hyperglycemia ratio, *BMI* body mass index, *COPD* chronic obstructive pulmonary disease, *EuroSCORE II* European system for cardiac operative risk evaluation, *Hs-CRP* high sensitivity C reactive protein, *LDL-C* low-density lipoprotein cholesterol, *HDL-C* high-density lipoprotein cholesterol, *NT-proBNP* N-terminal brain natriuretic peptide, *cTnI* cardiac troponin I, *LVEF* left ventricular ejection fraction, *NYHA* New York Heart Association, *ACEI* angiotensin-converting enzyme inhibitors, *ARB* angiotensin receptor blockers.

Supplementary Table 3 Baseline information on occurrence and absence of MACE

|  | No MACE  (n=518) | MACE  (n=64) | p value |
| --- | --- | --- | --- |
| Age, years | 75.28 ± 7.57 | 77.26 ± 6.03 | 0.044 |
| Male, % | 295 (56.95%) | 45 (70.31%) | 0.041 |
| BMI, kg/m^2^ | 23.61 ± 3.55 | 23.39 ± 4.35 | 0.642 |
| EuroSCORE II, % | 0.03 (0.02–0.05) | 0.04 (0.02–0.06) | 0.025 |
| NYHA class ≥ III, % | 382 (73.75%) | 54 (84.38%) | 0.064 |
| Smoking status, % |  |  | 0.374 |
| Never | 318 (61.39%) | 34 (53.12%) |  |
| Ex-smoker | 151 (29.15%) | 24 (37.50%) |  |
| Current | 49 (9.46%) | 6 (9.38%) |  |
| Hypertension, % | 325 (62.74%) | 39 (60.94%) | 0.779 |
| Hyperlipemia, % | 331 (63.90%) | 31 (48.44%) | 0.016 |
| Coronary heart disease, % | 216 (41.70%) | 35 (54.69%) | 0.048 |
| Previous myocardial infarction, % | 38 (7.34%) | 8 (12.50%) | 0.149 |
| Previous coronary revascularization, % | 83 (16.02%) | 13 (20.31%) | 0.383 |
| Chronic heart failure, % | 196 (37.84%) | 29 (45.31%) | 0.247 |
| Atrial fibrillation, % | 82 (15.83%) | 16 (25.00%) | 0.064 |
| Peripheral arterial disease, % | 82 (15.83%) | 10 (15.62%) | 0.966 |
| Previous valvular intervention, % | 11 (2.12%) | 3 (4.69%) | 0.207 |
| Previous stroke, % | 58 (11.20%) | 13 (20.31%) | 0.036 |
| COPD, % | 52 (10.04%) | 9 (14.06%) | 0.321 |
| Chronic kidney disease, % | 43 (8.30%) | 7 (10.94%) | 0.478 |
| Diabetes mellitus, % | 169 (32.63%) | 23 (35.94%) | 0.595 |
| Systolic blood pressure, mmHg | 128.85 ± 21.79 | 124.05 ± 18.95 | 0.093 |
| Diastolic blood pressure, mmHg | 69.21 ± 12.20 | 71.44 ± 13.06 | 0.172 |
| Heart rate, beats/min | 75.27 ± 12.87 | 74.48 ± 11.83 | 0.642 |
| Hemoglobin, g/L | 128.43 ± 18.49 | 123.94 ± 17.79 | 0.066 |
| Platelet, 10^9^/L | 192.17 ± 61.84 | 182.41 ± 45.86 | 0.222 |
| Albumin, g/L | 40.34 ± 4.01 | 38.91 ± 4.07 | 0.007 |
| Uric acid, μmol/L | 413.01 ± 135.62 | 414.68 ± 160.08 | 0.927 |
| eGFR, ml/min/1.73m^2^ | 62.75 ± 18.00 | 64.92 ± 17.10 | 0.359 |
| Hs-CRP, mg/L | 1.71 (1.00–4.43) | 1.69 (0.81–6.71) | 0.839 |
| Lipoprotein (a), mg/L | 199.50 (78.98–467.86) | 241.41 (101.88–679.65) | 0.128 |
| Triglyceride, mmol/L | 1.32 ± 0.88 | 1.20 ± 0.44 | 0.291 |
| Total cholesterol, mmol/L | 4.24 ± 1.12 | 4.15 ± 0.99 | 0.554 |
| LDL-C, mmol/L | 2.54 ± 0.96 | 2.57 ± 0.92 | 0.789 |
| HDL-C, mmol/L | 1.26 ± 0.38 | 1.20 ± 0.32 | 0.240 |
| NT-proBNP, pg/mL | 1929.00 (820.48–5329.25) | 2528.00 (1436.50–5683.25) | 0.142 |
| cTnI ratio | 0.74 (0.36–1.52) | 0.73 (0.44–1.32) | 0.897 |
| LVEF, % | 55.30 ± 13.65 | 52.21 ± 14.63 | 0.09 |
| Left atrial diameter, mm | 41.67 ± 6.27 | 44.03 ± 5.85 | 0.003 |
| Left ventricular diastolic diameter, mm | 51.40 ± 8.10 | 53.31 ± 8.66 | 0.077 |
| Moderate-to-severe mitral regurgitation, % | 102 (19.69%) | 20 (31.25%) | 0.032 |
| ACEI/ARB, % | 106 (20.46%) | 12 (18.75%) | 0.748 |
| β-blocker, % | 370 (71.43%) | 40 (62.50%) | 0.140 |
| Aspirin, % | 390 (75.29%) | 43 (67.19%) | 0.161 |
| P2Y12 inhibitor, % | 426 (82.24%) | 45 (70.31%) | 0.022 |
| Anticoagulant, % | 44 (8.49%) | 5 (7.81%) | 0.853 |
| Statin, % | 397 (76.64%) | 42 (65.62%) | 0.053 |
| Glucose-lowering therapy, % |  |  |  |
| Diet control | 9 (7.14%) | 2 (13.33%) | 0.698 |
| Oral hypoglycemic drugs | 82 (65.08%) | 9 (60.00%) |  |
| Insulin | 35 (27.78%) | 4 (26.67%) |  |
| Post-procedure mean gradient, mmHg | 12.42 ± 5.89 | 11.73 ± 4.08 | 0.361 |
| Moderate-to-severe perivalvular leakage, % | 12 (2.32%) | 4 (6.25%) | 0.069 |

*SHR* stress hyperglycemia ratio, *BMI* body mass index, *COPD* chronic obstructive pulmonary disease, *EuroSCORE II* European system for cardiac operative risk evaluation, *Hs-CRP* high sensitivity C reactive protein, *LDL-C* low-density lipoprotein cholesterol, *HDL-C* high-density lipoprotein cholesterol, *NT-proBNP* N-terminal brain natriuretic peptide, *cTnI* cardiac troponin I, *LVEF* left ventricular ejection fraction, *NYHA* New York Heart Association, *ACEI* angiotensin-converting enzyme inhibitors, *ARB* angiotensin receptor blockers.

Supplementary Table 4 Univariate analysis for all-cause mortality

|  | HR (95% CI) | *P* value |
| --- | --- | --- |
| Age (per year) | 1.02 (1.00, 1.05) | 0.081 |
| Female sex | 0.65 (0.45, 0.94) | 0.024 |
| BMI (per kg/m^2^ increase) | 0.98 (0.93, 1.02) | 0.347 |
| EuroSCORE II (per 0.01 score increase) | 1.05 (1.02, 1.07) | 0.001 |
| NYHA class ≥ III | 1.27 (0.82, 1.97) | 0.278 |
| Smoking ever | 1.31 (0.93,1.85) | 0.127 |
| Hypertension | 1.30 (0.90, 1.88) | 0.164 |
| Hyperlipemia | 0.87 (0.61, 1.24) | 0.448 |
| Coronary heart disease | 1.25 (0.89, 1.77) | 0.199 |
| Previous myocardial infarction | 0.90 (0.47, 1.72) | 0.754 |
| Previous coronary revascularization | 1.31 (0.85, 2.03) | 0.223 |
| Chronic heart failure | 1.21 (0.85, 1.72) | 0.299 |
| Atrial fibrillation | 1.41 (0.92, 2.15) | 0.115 |
| Peripheral arterial disease | 1.19 (0.75, 1.89) | 0.460 |
| Previous valvular intervention | 1.08 (0.40, 2.93) | 0.882 |
| Previous stroke | 1.73 (0.64, 4.69) | 0.282 |
| COPD | 1.65 (1.05, 2.59) | 0.030 |
| Chronic kidney disease | 2.35 (1.45, 3.79) | 0.001 |
| Diabetes mellitus | 1.05 (0.74, 1.51) | 0.772 |
| Systolic blood pressure (per mmHg increase) | 1.00 (0.99, 1.01) | 0.782 |
| Diastolic blood pressure (per mmHg increase) | 1.01 (0.99, 1.02) | 0.460 |
| Heart rate (per beats/min increase) | 1.01 (0.99, 1.02) | 0.282 |
| Hemoglobin (per g/L increase) | 0.99 (0.98, 1.00) | 0.191 |
| Platelet (per 10^9^/L increase) | 1.00 (1.00, 1.00) | 0.958 |
| Albumin (per g/L increase) | 0.92 (0.88, 0.96) | <0.001 |
| Uric acid (per μmol/L increase) | 1.00 (1.00, 1.00) | 0.099 |
| eGFR (per ml/min/1.73m^2^ increase) | 0.99 (0.98, 1.00) | 0.045 |
| Hs-CRP (per mg/L increase) | 1.06 (1.02, 1.11) | 0.002 |
| Lipoprotein (a) (per mg/L increase) | 1.00 (1.00, 1.00) | 0.046 |
| Triglyceride (per mmol/L increase) | 1.11 (0.93, 1.31) | 0.249 |
| Total cholesterol (per mmol/L increase) | 1.06 (0.90, 1.25) | 0.480 |
| LDL-C (per mmol/L increase) | 1.13 (0.95, 1.35) | 0.176 |
| HDL-C (per mmol/L increase) | 0.61 (0.37, 1.02) | 0.058 |
| NT-proBNP (per pg/mL increase) | 1.00 (1.00, 1.00) | 0.270 |
| cTnI ratio (per 1 increase) | 1.00 (1.00, 1.00) | 0.745 |
| LVEF (per 1% increase) | 1.00 (0.99, 1.01) | 0.966 |
| Left atrial diameter (per mm increase) | 1.05 (1.02, 1.08) | <0.001 |
| Left ventricular diastolic diameter (per mm increase) | 1.00 (0.98, 1.02) | 1.000 |
| Moderate-to-severe mitral regurgitation | 1.59 (1.08, 2.34) | 0.018 |
| ACEI/ARB | 1.38 (0.93, 2.07) | 0.113 |
| β-blocker | 1.11 (0.75, 1.63) | 0.597 |
| Aspirin | 1.14 (0.78, 1.67) | 0.507 |
| P2Y12 inhibitor | 0.84 (0.55, 1.28) | 0.412 |
| Anticoagulant | 0.93 (0.47, 1.84) | 0.843 |
| Statin | 0.74 (0.51, 1.07) | 0.105 |
| Glucose-lowering therapy |  |  |
| Diet control | Ref. |  |
| Oral hypoglycemic drugs | 0.60 (0.22, 1.61) | 0.308 |
| Insulin | 0.68 (0.23, 2.00) | 0.480 |
| Post-procedure mean gradient (per mmHg increase) | 0.99 (0.96, 1.02) | 0.433 |
| Moderate-to-severe perivalvular leakage | 2.76 (1.28, 5.93) | 0.009 |

*HR* hazard ratio, *CI* confidence interval, *SHR* stress hyperglycemia ratio, *BMI* body mass index, *COPD* chronic obstructive pulmonary disease, *EuroSCORE II* European system for cardiac operative risk evaluation, *Hs-CRP* high sensitivity C reactive protein, *LDL-C* low-density lipoprotein cholesterol, *HDL-C* high-density lipoprotein cholesterol, *NT-proBNP* N-terminal brain natriuretic peptide, *cTnI* cardiac troponin I, *LVEF* left ventricular ejection fraction, *NYHA* New York Heart Association, *ACEI* angiotensin-converting enzyme inhibitors, *ARB* angiotensin receptor blockers.

Supplementary Table 5 Univariate analysis for cardiovascular mortality or readmission for heart failure

|  | HR (95% CI) | *P* value |
| --- | --- | --- |
| Age (per year) | 1.02 (0.98, 1.06) | 0.306 |
| Female sex | 0.77 (0.46, 1.30) | 0.329 |
| BMI (per kg/m^2^ increase) | 0.97 (0.91, 1.04) | 0.411 |
| EuroSCORE II (per 0.01 score increase) | 1.06 (1.02, 1.09) | 0.001 |
| NYHA class ≥ III | 1.48 (0.76, 2.85) | 0.246 |
| Smoking ever | 1.10 (0.66, 1.84) | 0.713 |
| Hypertension | 1.21 (0.71, 2.06) | 0.473 |
| Hyperlipemia | 0.74 (0.45, 1.24) | 0.256 |
| Coronary heart disease | 1.79 (1.08, 2.98) | 0.025 |
| Previous myocardial infarction | 1.60 (0.75, 3.41) | 0.226 |
| Previous coronary revascularization | 1.49 (0.80, 2.77) | 0.206 |
| Chronic heart failure | 1.34 (0.80, 2.25) | 0.260 |
| Atrial fibrillation | 2.19 (1.24, 3.85) | 0.007 |
| Peripheral arterial disease | 1.37 (0.70, 2.65) | 0.356 |
| Previous valvular intervention | 3.86 (1.54, 9.68) | 0.004 |
| Previous stroke | - | - |
| COPD | 1.19 (0.56, 2.50) | 0.654 |
| Chronic kidney disease | 1.74 (0.79, 3.84) | 0.172 |
| Diabetes mellitus | 0.93 (0.55, 1.58) | 0.782 |
| Systolic blood pressure (per mmHg increase) | 0.99 (0.98, 1.00) | 0.160 |
| Diastolic blood pressure (per mmHg increase) | 1.01 (0.99, 1.03) | 0.460 |
| Heart rate (per beats/min increase) | 1.00 (0.98, 1.03) | 0.701 |
| Hemoglobin (per g/L increase) | 1.00 (0.98, 1.01) | 0.553 |
| Platelet (per 10^9^/L increase) | 1.00 (1.00, 1.00) | 0.955 |
| Albumin (per g/L increase) | 0.92 (0.86, 0.98) | 0.010 |
| Uric acid (per μmol/L increase) | 1.00 (1.00, 1.00) | 0.894 |
| eGFR (per ml/min/1.73m^2^ increase) | 1.00 (0.98, 1.01) | 0.743 |
| Hs-CRP (per mg/L increase) | 1.06 (1.00, 1.12) | 0.052 |
| Lipoprotein (a) (per mg/L increase) | 1.00 (1.00, 1.00) | 0.064 |
| Triglyceride (per mmol/L increase) | 0.94 (0.63, 1.39) | 0.742 |
| Total cholesterol (per mmol/L increase) | 0.98 (0.77, 1.25) | 0.866 |
| LDL-C (per mmol/L increase) | 1.16 (0.90, 1.50) | 0.241 |
| HDL-C (per mmol/L increase) | 0.75 (0.37, 1.51) | 0.421 |
| NT-proBNP (per pg/mL increase) | 1.00 (1.00, 1.00) | 0.226 |
| cTnI ratio (per 1 increase) | 1.00 (1.00, 1.00) | 0.847 |
| LVEF (per 1% increase) | 0.98 (0.97, 1.00) | 0.070 |
| Left atrial diameter (per mm increase) | 1.07 (1.03, 1.11) | 0.001 |
| Left ventricular diastolic diameter (per mm increase) | 1.02 (0.98, 1.05) | 0.314 |
| Moderate-to-severe mitral regurgitation | 1.89 (1.10, 3.27) | 0.022 |
| ACEI/ARB | 1.09 (0.58, 2.07) | 0.786 |
| β-blocker | 0.63 (0.38, 1.06) | 0.083 |
| Aspirin | 0.99 (0.57, 1.70) | 0.962 |
| P2Y12 inhibitor | 0.67 (0.38, 1.20) | 0.178 |
| Anticoagulant | 1.15 (0.46, 2.89) | 0.761 |
| Statin | 0.76 (0.44, 1.30) | 0.314 |
| Glucose-lowering therapy |  |  |
| Diet control | Ref. |  |
| Oral hypoglycemic drugs | 0.99 (0.12, 8.13) | 0.994 |
| Insulin | 1.15 (0.13, 10.46) | 0.902 |
| Post-procedure mean gradient (per mmHg increase) | 0.99 (0.94, 1.04) | 0.667 |
| Moderate-to-severe perivalvular leakage | 5.66 (2.41, 13.25) | <0.001 |

*HR* hazard ratio, *CI* confidence interval, *SHR* stress hyperglycemia ratio, *BMI* body mass index, *COPD* chronic obstructive pulmonary disease, *EuroSCORE II* European system for cardiac operative risk evaluation, *Hs-CRP* high sensitivity C reactive protein, *LDL-C* low-density lipoprotein cholesterol, *HDL-C* high-density lipoprotein cholesterol, *NT-proBNP* N-terminal brain natriuretic peptide, *cTnI* cardiac troponin I, *LVEF* left ventricular ejection fraction, *NYHA* New York Heart Association, *ACEI* angiotensin-converting enzyme inhibitors, *ARB* angiotensin receptor blockers.

Supplementary Table 6 Univariate analysis for MACE

|  | HR (95% CI) | *P* value |
| --- | --- | --- |
| Age (per year) | 1.03 (0.99, 1.07) | 0.170 |
| Female sex | 0.60 (0.35, 1.03) | 0.064 |
| BMI (per kg/m^2^ increase) | 0.98 (0.91, 1.04) | 0.468 |
| EuroSCORE II (per 0.01 score increase) | 1.05 (1.01, 1.09) | 0.014 |
| NYHA class ≥ III | 1.84 (0.93, 3.65) | 0.080 |
| Smoking ever | 1.34 (0.82, 2.18) | 0.248 |
| Hypertension | 0.99 (0.60, 1.65) | 0.980 |
| Hyperlipemia | 0.61 (0.37, 1.00) | 0.050 |
| Coronary heart disease | 1.65 (1.01, 2.71) | 0.047 |
| Previous myocardial infarction | 1.44 (0.68, 3.05) | 0.341 |
| Previous coronary revascularization | 1.34 (0.73, 2.47) | 0.346 |
| Chronic heart failure | 1.33 (0.80, 2.21) | 0.264 |
| Atrial fibrillation | 1.97 (1.11, 3.49) | 0.021 |
| Peripheral arterial disease | 1.13 (0.57, 2.24) | 0.726 |
| Previous valvular intervention | 1.67 (0.52, 5.37) | 0.388 |
| Previous stroke | 27.78 (14.11, 54.69) | <0.001 |
| COPD | 1.33 (0.65, 2.70) | 0.430 |
| Chronic kidney disease | 1.59 (0.72, 3.51) | 0.249 |
| Diabetes mellitus | 1.02 (0.61, 1.71) | 0.931 |
| Systolic blood pressure (per mmHg increase) | 1.00 (0.98, 1.01) | 0.559 |
| Diastolic blood pressure (per mmHg increase) | 1.02 (1.00, 1.04) | 0.136 |
| Heart rate (per beats/min increase) | 1.00 (0.98, 1.02) | 0.966 |
| Hemoglobin (per g/L increase) | 0.99 (0.98, 1.01) | 0.414 |
| Platelet (per 10^9^/L increase) | 1.00 (0.99, 1.00) | 0.472 |
| Albumin (per g/L increase) | 0.92 (0.87, 0.98) | 0.013 |
| Uric acid (per μmol/L increase) | 1.00 (1.00, 1.00) | 0.903 |
| eGFR (per ml/min/1.73m^2^ increase) | 1.00 (0.99, 1.02) | 0.774 |
| Hs-CRP (per mg/L increase) | 1.03 (0.97, 1.09) | 0.403 |
| Lipoprotein (a) (per mg/L increase) | 1.00 (1.00, 1.00) | 0.007 |
| Triglyceride (per mmol/L increase) | 0.81 (0.51, 1.28) | 0.357 |
| Total cholesterol (per mmol/L increase) | 0.97 (0.76, 1.23) | 0.801 |
| LDL-C (per mmol/L increase) | 1.05 (0.81, 1.37) | 0.700 |
| HDL-C (per mmol/L increase) | 0.80 (0.41, 1.58) | 0.519 |
| NT-proBNP (per pg/mL increase) | 1.00 (1.00, 1.00) | 0.433 |
| cTnI ratio (per 1 increase) | 1.00 (1.00, 1.00) | 0.774 |
| LVEF (per 1% increase) | 0.99 (0.97, 1.01) | 0.189 |
| Left atrial diameter (per mm increase) | 1.06 (1.02, 1.10) | 0.002 |
| Left ventricular diastolic diameter (per mm increase) | 1.02 (0.99, 1.06) | 0.128 |
| Moderate-to-severe mitral regurgitation | 1.99 (1.16, 3.39) | 0.012 |
| ACEI/ARB | 1.02 (0.54, 1.93) | 0.940 |
| β-blocker | 0.74 (0.45, 1.24) | 0.255 |
| Aspirin | 1.02 (0.60, 1.75) | 0.929 |
| P2Y12 inhibitor | 0.58 (0.33, 1.01) | 0.053 |
| Anticoagulant | 1.13 (0.45, 2.83) | 0.792 |
| Statin | 0.64 (0.38, 1.09) | 0.100 |
| Glucose-lowering therapy |  |  |
| Diet control | Ref. |  |
| Oral hypoglycemic drugs | 0.55 (0.12, 2.59) | 0.452 |
| Insulin | 0.57 (0.10, 3.16) | 0.524 |
| Post-procedure mean gradient (per mmHg increase) | 0.98 (0.93, 1.03) | 0.397 |
| Moderate-to-severe perivalvular leakage | 3.31 (1.20, 9.17) | 0.021 |

*HR* hazard ratio, *CI* confidence interval, *SHR* stress hyperglycemia ratio, *BMI* body mass index, *COPD* chronic obstructive pulmonary disease, *EuroSCORE II* European system for cardiac operative risk evaluation, *Hs-CRP* high sensitivity C reactive protein, *LDL-C* low-density lipoprotein cholesterol, *HDL-C* high-density lipoprotein cholesterol, *NT-proBNP* N-terminal brain natriuretic peptide, *cTnI* cardiac troponin I, *LVEF* left ventricular ejection fraction, *NYHA* New York Heart Association, *ACEI* angiotensin-converting enzyme inhibitors, *ARB* angiotensin receptor blockers.

Supplementary Table 7 HRs (95% CIs) for different endpoints of SHR after excluding those with hemoglobin ＜ 100g/L (n = 564)

|  | Per 0.1 increase in SHR | High vs low SHR* |
| --- | --- | --- |
| All-cause mortality |  |  |
| Model 2 | 1.13 (1.04–1.22) | 1.51 (1.01–2.27) |
| Cardiovascular mortality or readmission for heart failure |  |  |
| Model 2 | 1.14 (1.03–1.25) | 2.08 (1.20–3.61) |
| MACE |  |  |
| Model 2 | 1.14 (1.03–1.26) | 1.78 (1.03–3.07) |

*The dichotomy of SHR (0.944) was determined by the maximally selected rank statistics.

*SHR* stress hyperglycemia ratio, *MACE* major adverse cardiovascular events, *HR* hazard ratio, *CI* confidence interval

Supplementary Table 8 HRs (95% CIs) for different endpoints of SHR after excluding those with eGFR < 30 ml/min/m^2^ (n = 559)

|  | Per 0.1 increase in SHR | High vs low SHR* |
| --- | --- | --- |
| All-cause mortality |  |  |
| Model 2 | 1.12 (1.04–1.21) | 1.51 (1.01–2.27) |
| Cardiovascular mortality or readmission for heart failure |  |  |
| Model 2 | 1.14 (1.03–1.25) | 2.08 (1.20–3.61) |
| MACE |  |  |
| Model 2 | 1.13 (1.02–1.25) | 1.77 (1.02–3.07) |

*The dichotomy of SHR (0.944) was determined by the maximally selected rank statistics.

*SHR* stress hyperglycemia ratio, *MACE* major adverse cardiovascular events, *HR* hazard ratio, *CI* confidence interval
